# A Novel Homomorphic Approach for Preserving Privacy of Patient Data in Telemedicine

**DOI:** 10.3390/s22124432

**Published:** 2022-06-11

**Authors:** Yasir Iqbal, Shahzaib Tahir, Hasan Tahir, Fawad Khan, Saqib Saeed, Abdullah M. Almuhaideb, Adeel M. Syed

**Affiliations:** 1 Department of Information Security, College of Signals, National University of Sciences and Technology (NUST), Rawalpindi 46000, Pakistan; yasiriqbal.engr@gmail.com (Y.I.); fawadkhan@mcs.edu.pk (F.K.); 2 School of Electrical Engineering and Computer Science, National University of Sciences and Technology (NUST), Islamabad 44000, Pakistan; hasan.tahir@seecs.edu.pk; 3 SAUDI ARAMCO Cybersecurity Chair, Department of Computer Information Systems, College of Computer Science and Information Technology, Imam Abdulrahman Bin Faisal University, Dammam 34212, Saudi Arabia; sbsaed@iau.edu.sa; 4 SAUDI ARAMCO Cybersecurity Chair, Department of Networks and Communications, College of Computer Science and Information Technology, Imam Abdulrahman Bin Faisal University, Dammam 34212, Saudi Arabia; amalmuhaideb@iau.edu.sa; 5 Department of Software Engineering, Bahria University, Islamabad 44000, Pakistan; adeel@bahria.edu.pk

**Keywords:** fully homomorphic encryption (FHE), cloud, searchable encryption, phoneme/audio searching

## Abstract

Globally, the surge in disease and urgency in maintaining social distancing has reawakened the use of telemedicine/telehealth. Amid the global health crisis, the world adopted the culture of online consultancy. Thus, there is a need to revamp the conventional model of the telemedicine system as per the current challenges and requirements. Security and privacy of data are main aspects to be considered in this era. Data-driven organizations also require compliance with regulatory bodies, such as HIPAA, PHI, and GDPR. These regulatory compliance bodies must ensure user data privacy by implementing necessary security measures. Patients and doctors are now connected to the cloud to access medical records, e.g., voice recordings of clinical sessions. Voice data reside in the cloud and can be compromised. While searching voice data, a patient’s critical data can be leaked, exposed to cloud service providers, and spoofed by hackers. Secure, searchable encryption is a requirement for telemedicine systems for secure voice and phoneme searching. This research proposes the secure searching of phonemes from audio recordings using fully homomorphic encryption over the cloud. It utilizes IBM’s homomorphic encryption library (HElib) and achieves indistinguishability. Testing and implementation were done on audio datasets of different sizes while varying the security parameters. The analysis includes a thorough security analysis along with leakage profiling. The proposed scheme achieved higher levels of security and privacy, especially when the security parameters increased. However, in use cases where higher levels of security were not desirous, one may rely on a reduction in the security parameters.

## 1. Introduction

**Figure 1:**
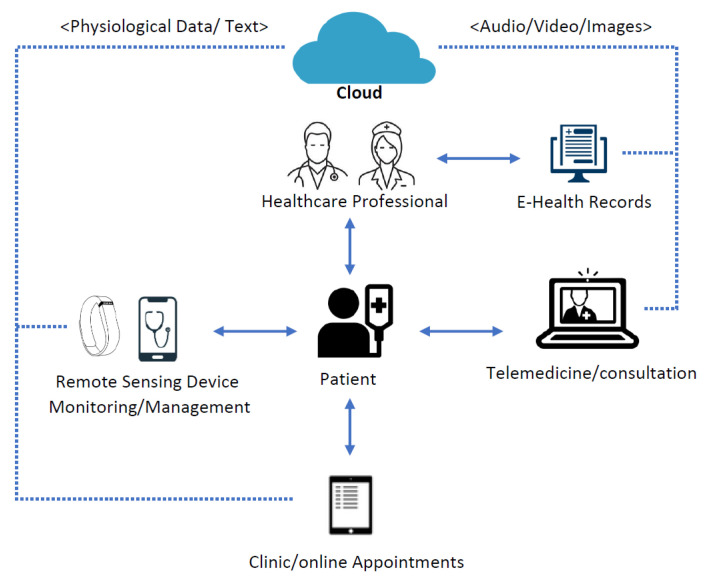
Overview of telemedicine architecture.

‘Telemedicine’ was a phrase coined literally to mean ‘remote healing’ in the 1970s [1]. Through the use of communication technology, telemedicine could assist health practitioners in obtaining medical information about patients. To distinguish between telehealth and telemedicine, the author in [2] offer the following definition: ‘The telemedicine is a part of telehealth that specifically offers clinical services remotely.’ The word ‘telehealth’ refers to a wide range of technologies that improve healthcare delivery systems and provide non-clinical services, such as medical education, training, administrative meetings, etc. Researchers believe that telemedicine can solve many problems when a global health crises, such as a pandemic or epidemic, is at hand, but to our knowledge, its potential has not been widely explored due to security concerns associated with telemedicine. Moreover, the increasing number of patients in hospitals, remote healthcare for the elderly, and pandemics have highlighted the need for telemedicine services. Many people are isolated during hospitalization or while quarantining at home, so telemedicine services must be adopted. According to the researchers, simple e-health solutions may allow seriously ill patients to communicate with one another and acquire relevant health information more quickly. Figure 1 describes the detailed model of the telemedicine system in the current scenario. In this architecture, our focus is on a patient-centric approach. The e-health records of the patients from all digital gadgets are stored in the cloud. The e-health records are in the form of audio/video/images. All authorized stakeholders of the system can access these data from the cloud.

The surge in the adoption of cloud computing in the telehealth sector raises many security and privacy challenges. As per the report [3], there was a 40.63% increase in data breaches of healthcare records in February 2021. A patient died due to a ransomware cyberattack in Germany that disrupted the hospital facility system [4].

The communication modes in telemedicine between patients and doctors are video links, voice calls, voice notes, and text messages. Multiple third-party clinical service applications [5,6] are now fully operational, and many new companies have been formed to deliver remote healthcare services. These third-party services provide features to facilitate communication and store the recordings of clinical sessions on the cloud. Those clinical services or electronic health platforms must comply with regulatory bodies, e.g., the Health Insurance Portability and Accountability Act (HIPAA). Due to this, data security and privacy are crucial while storing data off-premise. The data residing in cloud service providers (CSPs) invite cyber threats. Besides common security threats against confidentiality, integrity, and availability, other threats related to CSPs are co-tenancy, cross-VM attacks using side channels, and loss of physical controls [7,8,9]. The design goals that are being achieved through this research are discussed below.

### 1.1. Design Goals

- Data confidentiality—the conventional mechanism for achieving confidentiality is storing data in encrypted form [10]. Therefore, data must be confidential before uploading to the cloud.
- Searching capability—there is a need for a mechanism to search over the cloud without decryption, and a need for retrieving or downloading only the desired file.
- Authorized person searching—only the authorized person who has keys should generate the trapdoor and search requests.
- Privacy-preserving—while sending the search query request to the server, the adversary should not know any information about the query. The trapdoor should be probabilistic, so the ciphertext will differ each time the request is made.
- Search pattern hiding—the outcome of the searching should be probabilistic, such that a keyword being searched repeatedly should be indistinguishable, hence hiding the search pattern.
- Cloud deployable—the searchable encryption scheme should be deployable in a client–server architecture using any cloud service, and it should be able to integrate into an existing enterprise network.

### 1.2. Contributions

The following contributions are made through this research:

- This research focuses on phonemes/audio searching over the cloud without decrypting the audio files. As discussed earlier, the audio files of conversations between health professionals and patients are usually stored in the cloud.
- This research proposes a mechanism to search for the desired phoneme keyword among the encrypted audio files in the cloud. This proposed scheme does not need to maintain any index table. Thus, it avoids several other data leakages and reduces the attack surface.
- For this purpose, the medical voice dataset was used; the proposed mechanism was deployed and tested in a public cloud platform, “Contabo”, using the client/server architecture. This paper also presents a comprehensive security and performance analysis of the proposed scheme, which verifies the fulfillment of privacy preservation.

The organization of this paper is as follows: Section 2 discusses the related articles, and Section 3 presents the preliminaries and the system model. Section 4 presents the security definitions for the proposed scheme. Section 5 presents the proposed phoneme searching framework. Section 6 performs the security analysis of the proposed scheme. Section 7 illustrates the performance analysis, and Section 8 discusses the scheme. The conclusions and future work are presented in Section 9 of the paper.

## 2. Literature Review

Our literature review is divided into three sections. The first section sheds light on secure audio/voice processing, the second section focuses on privacy preservation in telemedicine, and the third section explores the existing searchable encryption approaches.

### 2.1. Secure Voice Processing

Research has been conducted on secure voice processing using homomorphic encryption. The paper [11] proposed a novel approach based on the Newton iterative method to redesign the mechanism of the speaker verification system using fully homomorphic encryption. This way, the privacy of voice data is achieved while the efficiency is increased. The proposed algorithm is tested on a speech dataset using SEAL and TenSEAL libraries. The dataset description shows that each recording lasts for 3 s maximum, which is very small and suitable for the verification systems (biometric). However, when we talk about searching across files of variable lengths, the performances may vary and the usability of the system at a large scale may give rise to usability concerns. A paper published by Microsoft [12] proposed a mechanism for the detection of a suicidal idea from phone conversations while preserving privacy using a homomorphic evaluation of neural networks. This research work was also evaluated using the SEAL library. However, it lacked a discussion of the proposed scheme’s security definitions or security analysis. The authors in [13] presented research on the privacy-preserving of phonetic searching over voice data. The proposed scheme is based on index-based searching, while the encryption is also deterministic, which increases the security leakages. The paper [14] provides detailed insights into privacy-preserving speech data.

### 2.2. Privacy-Preserving Approaches in Telemedicine

Several different approaches have been proposed in the literature to make telemedicine secure and ‘privacy-preserving’. For instance, Zhang, in [15], proposed a solution for privacy preservation in electronic health records based on blockchain technology. Furthermore, their proposed system uses pairing-based cryptography to prevent the EHR data from being altered. The paper presented the privacy-preserving method with blockchain technology following five phases to achieve privacy in e-health. For example, they used a signature with a password for authentication, a secure key exchange protocol for key sharing, and the signature before performing encryption for electronic health record (EHR) generation, hashing with a transaction signature for tamper-proof record generation, and a smart contracts method for secure payments. An article [4] published in 2020 during a pandemic situation focused on the information and privacy of telemedicine systems. This article emphasized using cybersecurity measures with modern technologies, e.g., artificial intelligence and the internet of things (IoT) to detect cyberattacks.

The authors in [16] proposed a solution for electronic health records (EHRs), including telemedicine. The research goal was to preserve the privacy that utilizes hyperledger technology and the identity mixer suite. The authorized healthcare professionals can store health records and allow them to access the documents. Another paper [17] addressed privacy and authentication-related issues in telemedicine. It proposed a digital watermarking method to deal with authentication issues.

The researchers of [18] proposed an integrated system that protects the privacy of different types of information regarding health systems, including telemedicine. This system combines medical information, such as e-prescriptions, smart cards, e-patient records, and fingerprint identification, and applies proxy and group signatures. This research aims to eliminate the paperwork and shift to digitization while protecting all information. However, this research still does not talk about preserving privacy while searching the data over the cloud or server.

The authors of [19] discussed the health data used in telemedicine and privacy issues related to access control mechanisms in hybrid clouds (when the data are uploaded to the cloud). They used the XACML method for access control in clouds that store healthcare data, so it needs a secure mechanism with privacy preservation. However, again, a challenge exists because the (massive amount of) data reside in the cloud, requiring secure searches of the data without decrypting them.

The authors in [10] presented a review on issues related to the security and privacy of electronic medical records (EMR) used in telemedicine. They also discussed the solutions that can solve those issues.

### 2.3. Searchable Encryption

The user desires to search specific data over the cloud while data remain encrypted in searchable encryption. For this purpose, the user generates the search query called trapdoor [20] or a search token [21] and sends it to the cloud server. With this search query, the server then searches using a searchable encryption scheme over the cloud and sends the relevant encrypted documents back to the user. For this purpose, different techniques for searchable encryption have been proposed. The first ranked-based approach in SE was proposed in [22]. Later, inverted index-based searching schemes were proposed for efficient searching, which also reduces leakages. Kamara et al. [23] enhanced index-based searching using homomorphic encryption. The authors encrypted the index pointer with HE to avoid leakages, but it still had a flaw because of the deterministic trapdoor.

The authors in [24] concealed the search pattern in order to guarantee trapdoor and keyword privacy. The authors used a particular type of additive homomorphic encryption to accomplish the conjunctive keyword searchable encryption. To meet the privacy aims of the strategy, they proposed two servers: a cloud server and an auxiliary server. They also used random polynomials to improve user security (by ensuring that the users only obtain the results they want). Their approach ensures a higher level of protection for cloud users. Their method runs a parallel search that is independent of the search index. Y. Wang et al. in [25] employed homomorphic encryption (HE) to perform effective multi-keyword retrieval. The authors used correlation ratings to provide the cloud user with reliable and ranked results. They optimized a homomorphic encryption technique to ensure secure document retrieval. They demonstrated that their method provides keyword privacy as well as quick retrieval of top-k documents. In comparison to previous ciphertext retrieval systems, their scheme provides data confidentiality and accurate results to cloud users. The research paper [26] proposed keyword searching for a multi-user environment based on homomorphic encryption. The authors claim to optimize the DGHV version of HE.

The authors in [27] proposed an enhanced version of k-nearest neighbors for searchable encryption (KNN-SE) to enhance efficiency and reduce leakages. The main features of this scheme are the support of arbitrary language, a multi-user environment, and improved security. Another scheme for multi-keyword searching based on a tree-based index was proposed in [28]. For the searching phase, the proposed system uses the depth-first search method to find the desired files. The authors in [29] employed the dual embedding space model (DESM) to produce accurate ranked search results. They also utilized a lightweight model for practical use. The creation of a DESM index provides faster retrieval of the ranked results. The authors used an upgraded k-NN to perform a multi-keyword ranked search on encrypted cloud data. In DESM, they used dimension-reduction to overcome the problem of index updates. They proved (by analysis) that their method is more efficient and has less overhead than other multi-keyword-ranked searchable approaches. Furthermore, more descriptions of proposed schemes, methods, and different topologies are briefly discussed in [20]. All proposed schemes discussed above are lacking in security and privacy. Some of them are based on an index that leaks data, some provide confidentiality but do not have search capabilities. Furthermore, all schemes do not achieve privacy preservation.

NOTE: The schemes discussed in our literature review achieve privacy preservation in their contexts, so we included them in Table 1. However, as per the definition of privacy-preserving in searchable encryption [30], privacy-preserving is achieved when the search pattern is preserved. Hence, not all of the schemes presented in the literature are privacy-preserving, as per the security definitions we follow.

## 3. System Model

**Figure 2:**
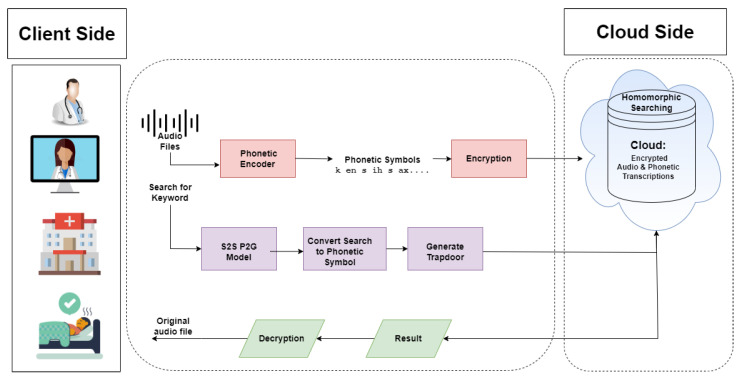
Architecture diagram of phonemes search.

In this use case, based on secure, searchable encryption, the proposed system searches phonemes over encrypted audio files. First, the audio file is passed through the encoder to generate its phonemes. The client encrypts the audio files in phonemic form and sends them to the server. The client also generates the trapdoor, the desired searching phonemes, and sends it to the server. The server then calculates homomorphically over encrypted phonemes and finds matched audio file(s) without decryption. The server then sends the searched results to the client. The client receives the file and decrypts it. The original file, consisting of phonemes, can be found after decrypting it, as shown in Figure 2. The notations used in this paper are given in Table 2.

### 3.1. Definitions

The proposed scheme comprises five phases, as defined below:

#### 3.1.1. KeysGen

($S_{k}$, $P_{k}$) ← GenSecKey*(hwt,p)*: This is a key generation algorithm run on the client-side. The algorithm takes a plaintext space modulus (*P*) and Hamming weight (*hwt*) as input and returns the public key $P_{k}$ and private key $S_{k}$.

#### 3.1.2. Encryption

($E_{Ph}$) ←*$Enc_{P}$(F,$P_{k}$)*: This is a probabilistic encryption algorithm run on the client-side. The algorithm takes plain text phonemes (*F*) and the public key $P_{k}$ as input and returns the ciphertext (C).

#### 3.1.3. Build Trapdoor

$T_{E}$← Build-Trap*(Ph,$P_{k}$)*: This is a probabilistic algorithm run on the client-side. The algorithm takes phonemes (*Ph*) and the public key ($P_{k}$). The algorithm returns a query or trapdoor ($T_{E}$).

#### 3.1.4. Searching

*R*← Search_Ph($T_{E},E_{Ph})$: This is an algorithm for searching phonemes homomorphically in the encrypted data on the server-side, which takes trapdoor ($T_{E}$) as input. It generates output encrypted search results (Enc_R).

#### 3.1.5. Decryption

X ←$Dec_{S_{k}}$(*R*,$S_{k}$): This is a probabilistic algorithm run on the client-side. It takes the input of the result (R) from the searching phase and recovers/decrypts the original phonemes file (X).

## 4. Security Definitions

In this section, definitions of keyword–trapdoor indistinguishability and trapdoor–document indistinguishability are discussed, which are taken from [30].

### 4.1. Definition 1–Keyword–Trapdoor Indistinguishability

When an adversary cannot distinguish between the keyword and its trapdoor, the cryptosystem is secure. Because of the probabilistic nature of the scheme, the ciphertext is different whenever it is encrypted. The adversary intercepts and fetches the trapdoor to identify its keyword (plaintext) in polynomial time. The adversary chooses a keyword and sends it to the challenger. The challenger then generates the trapdoor and sends it back to the adversary. This way, the number of trapdoors are generated in polynomial time. After that, the adversary has to select two keywords and gets its trapdoor from the challenger. The adversary then guesses and outputs.

Game 1. Adversary A and challenger C are two players in this game. Assume that there are phonemes *Ph* = $(Ph_{1}$, $Ph_{2}$, …, $Ph_{k})$ and *N* number of phoneme files *F* = ($F_{1}$,$F_{2}$, … $F_{N}$).

$$\begin{array}{l} \left(S_{k},P_{k},\right)\leftarrow GenSecKey\left(\mathrm{hwt},p\right)\\ E_{ph}\leftarrow Enc_{P}\left(P_{k},F_{N}\right)\\ for\;0<i<N:\\ \;T_{i}\leftarrow Build-Trap\left({Ph}_{i},P_{k}\right)\\ \;(s_{A},Q)\leftarrow A\left(s_{A},T_{i}\right)\\ toss\leftarrow \left\{0,1\right\};\\ \left(s_{A},Ph_{0},Ph_{1}\right)\leftarrow A\left(1^{\lambda }\right)\\ T_{Ph}{}_{toss}\leftarrow Build-Trap\left({Ph}_{toss},P_{k}\right)\\ {toss}^{\prime }\leftarrow A_{N+1}\left(s_{A},T_{Ph}{}_{toss}\right)\\ T_{Ph}{{}_{toss}}^{\prime }\leftarrow Build-Trap\left({Ph}_{j},P_{k}\right);j\in N\\ if{toss}^{\prime }=toss;\;output\;1;\\ otherwise\;output\;0 \end{array}$$

where $S_{A}$ is the state of the adversary *A*. The scheme is said to provide keyword–trapdoor indistinguishability, if the following holds true,

$$Pr\left[Kw\_Trap_{A}\left(\lambda \right)=1\right]\leq \frac{1}{2}+negl\left(\lambda \right)$$

The challenger begins by generating a set of encrypted documents. The three phases of this experiment are:

*Phase 1:* The adversary chooses a keyword of his own choice and sends it to the challenger. The challenger then generates the trapdoor of this keyword and sends it back to the adversary. This session continues between adversary and challenger for a polynomial time.
*Challenge Phase:* In this phase, the adversary chooses any two phonemes ($Ph_{1}$, $Ph_{2}$) and sends them to the challenger. Before generating a trapdoor, the challenger tosses a coin {0,1}. Then he generates a trapdoor against the keyword corresponding to the coin.
*Final Phase:* The adversary will now guess the keyword corresponding to the trapdoor. The challenger will see if the adversary’s guessed trapdoor matches with its keyword. If it is matched, then the adversary has won the game. As the adversary has to guess from the two options ($Ph_{1}$, $Ph_{2}$), and if the proposed scheme is probabilistic, the probability of winning the game is 0.5.

### 4.2. Definition 2–Trapdoor–Document Indistinguishability

In searchable encryption, the user generates the trapdoor against the keyword he wants to search for over encrypted data. After searching for a keyword, the output is a relevant encrypted document or file. If an adversary cannot distinguish between the trapdoor and its resulting file, the searchable encryption scheme is secure and termed to be privacy-preserving.

Game 2. Let us play a game between a challenger and an adversary. Assume that there are phonemes *Ph* = $(Ph_{1}$, $Ph_{2}$, …, $Ph_{k})$ and *N* number of phoneme files *F* = ($F_{1}$,$F_{2}$, … $F_{N}$). There are three phases of this game:

$$\begin{array}{l} \left(S_{k},P_{k},\right)\leftarrow GenSecKey\left(\mathrm{hwt},p\right)\\ E_{ph}\leftarrow Enc_{P}\left(P_{k},F_{N}\right)\\ for\;0<i<K:\\ \;(s_{A},T_{E})\leftarrow A\left(\left(s_{A},Ph_{1},Ph_{2},\ldots Ph_{k}\right)\right);F_{i}\leftarrow Search\_Ph\left(T_{E},E_{Ph},N\right)\\ toss\leftarrow \left\{0,1\right\};\\ \left(s_{A},T_{E_{0}},T_{E_{1}}\right)\leftarrow A\left(1^{\lambda }\right)\\ F^{\prime }\leftarrow Search\_Ph\left(T_{E}{}_{toss},E_{Ph},N\right)\\ toss^{\prime }\leftarrow A_{k+1}\left(s_{A},F^{\prime }\right);\\ T_{{toss}^{\prime }}\leftarrow Build-Trap\left(k_{qj},P_{k}\right);j\in N)\\ iftoss^{\prime }=toss;\;output\;1;\\ otherwise\;output\;0 \end{array}$$

where $S_{A}$ is the state of the adversary *A*. The scheme is said to provide trapdoor–document indistinguishability if the following holds true,

$$\begin{array}{c} Pr\left[Trap\_File_{A}\left(\lambda \right)=1\right]\leq \frac{1}{2}+negl\left(\lambda \right) \end{array}$$

The challenger begins by generating a set of encrypted files. The three phases of this experiment are:

*Phase 1:* The adversary chooses a keyword of his own choice and sends it to the challenger. The challenger then generates this keyword’s trapdoor and searches the relevant encrypted document. The challenger sends a trapdoor and encrypted file to the adversary. This session continues between the adversary and a challenger for a polynomial time.
*Challenge Phase:* In this phase, the adversary now chooses any two phonemes ($Ph_{1}^{\prime }$, $Ph_{2}^{\prime }$) and sends them to the challenger. The challenger tosses a coin (0,1) before generating a trapdoor and searching a relevant file. Then he generates two trapdoors and searches two corresponding files against those two keywords.
*Final Phase*: In this phase, the adversary now chooses any two Phonemes ($Ph_{1}^{\prime }$, $Ph_{2}^{\prime }$) and sends them to the challenger. The challenger tosses a coin (0,1) before generating a trapdoor and searching a relevant file. Then he generates two trapdoors and searches two corresponding files against those two keywords.
NOTE: The research paper [34] discusses the search pattern leakages. The author elaborates that the search pattern is accessible in deterministic schemes. However, even in the probabilistic nature of the scheme, the search pattern can still be disclosed using the entries in the index. Our scheme is based on fully homomorphic encryption, which does not require keeping an index table for searching the query. The searching was conducted based on a probabilistic trapdoor. Therefore the search pattern is not revealed in our scheme.

## 5. Proposed Phoneme Searching Framework

The proposed scheme ingests HElib operations presented in [35]. The proposed scheme is presented below:

### 5.1. Setup Phase

The setup phase takes several parameters as input and initializes the context. The context further generates the key pair (public and secret keys). The secret key then computes key switching matrices. The setup phase is shown in the Algorithm 1.
**Algorithm 1:** Setup Phase.(1) Input: *m,p,r,c,nthreads,bits*(2) *map.arg(input)*(3) Initialize the context // Object holding information about the scheme’s algebra*context(m,p,r,bits,c)*EA ← context() // Obtain the encrypted array of the context(4) Generate keys:keys $S_{k}$,$P_{k}$: GenSecKey(*context*)(5) Compute key-switching matrices:addSome1DMatrices($S_{k}$)(6) Output: *$S_{k}$, $P_{k}$*

### 5.2. Encryption Phase

The algorithm for the encryption phase conducts the encryption of multiple phoneme files using the public key. The algorithm first chooses one file and reads each line from the file that is converted into plain text representation by using a string to integer conversion. Finally, it performs encryption and saves to the vector. In this way, the algorithm will pick all the files one by one, and will encrypt. The encryption phase is shown in the Algorithm 2.
**Algorithm 2:** Encryption Phase.
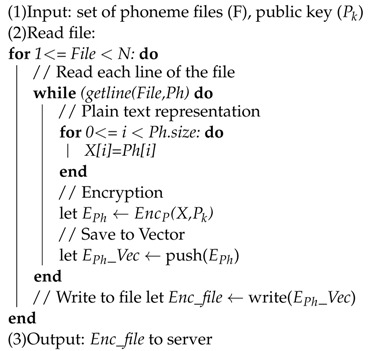


### 5.3. Build Trapdoor Phase

This algorithm generates a probabilistic trapdoor against the query keyword given by the client. It takes the query keyword and public key as input, converts the query into integer form, and performs encryption to generate a meaningful trapdoor. The generated trapdoor will be sent to the server for the searching phase. The trapdoor generation phase is shown in the Algorithm 3.
**Algorithm 3:** Build trapdoor phase.(1) Input: Query($k_{q},P_{k}$)(2) Convert query to a numerical vector:let *$N_{v}$*←*NumVect($k_{q}$)* //Encrypt the query$T_{E}$←*$Enc_{P}$($N_{v}$,$P_{k}$)*(3) Output: Transmit $T_{E}$ to server

### 5.4. Searching Phase

The algorithm for the searching phase is executed on the cloud side against the received trapdoor. The algorithm is designed in such a way that it searches for the desired keywords in all encrypted files. This algorithm will work when the client requests the query, as described in the build trapdoor phase. Once the trapdoor is received from the client, the cloud server will execute the search algorithm. The input arguments of this search algorithm are the trapdoor and encrypted files stored in the cloud. The algorithm picks one file at a time, reads each encrypted line (phoneme), and matches with the query homomorphically. Since we are performing a search on multiple files, the algorithm will search the required query on all the files stored in the cloud server. The main steps of the database searching phase in HElib include calculating the difference by subtraction, then power function, negating the ciphertext, and adding the constant. The setup phase is shown in the Algorithm 4.
**Algorithm 4:** Searching phase.
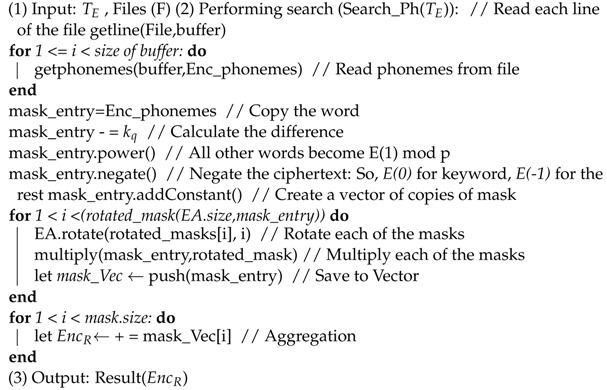


### 5.5. Decryption Phase

After searching for the desired result, the response is also probabilistically encrypted, which needs to be decrypted when received on the client-side. This algorithm is performed at the client-side, which takes the response from the cloud side as input. The decryption phase is shown in the Algorithm 5.
**Algorithm 5:** Decryption phase.(1) Input: result(*R*)(2) Decryption:X ←$Dec_{S_{k}}$(*R,$S_{k}$*)(3) Output: Search result in plaintext *(X)*

## 6. Security Analysis

Several previous research works on searchable encryption accepted the leakage of information either due to index-based searching or deterministic encryption [23,30,36]. Since our scheme is neither based on index nor deterministic encryption, it reduces the leakages compared to the state-of-the-art. The leakage profiles of the proposed scheme are presented below:

### 6.1. Leakage Profiling

Our goal was to analyze and present any leakage in our proposed model that may be encrypted/decrypted or meaningful/meaningless. The leakage profiling was performed against all five phases of the scheme presented in Section 4, and we considered all the artifacts evolving. The following leakages were determined:

(1) Leakage $L_{1}$: This leakage discusses the encrypted files stored in the cloud server. The data owner encrypts the audio files at the client end and uploads them to the cloud. The cloud server cannot distinguish the underlying data because the files are encrypted. However, the CSP will be able to know the size of the encrypted files. Although the encryption provided by HElib is probabilistic, each time, the file size is different. The cloud server cannot guess the original file but can only know the size. Moreover, CSP only can see the number of files stored in the cloud. Leakage $L_{1}$ is defined as:

$$\begin{array}{c} L_{1}=\left\{\begin{array}{c} E_{Audio},\left(number\;of\;E_{Audio}\right),\left(size\;of\;E_{Audio}\right) \end{array}\right\} \end{array}$$

(2) Leakage $L_{2}$: This leakage focuses on the trapdoor generated at the client-side and sent to the cloud. The encryption is performed on the query and generates the trapdoor. Again, the encryption is probabilistic, so the trapdoor is different and reveals no information to the cloud server about the phoneme being searched. Leakage $L_{2}$ is defined as:

$$\begin{array}{c} L_{2}=\left\{\begin{array}{c} Enc_{P_{k}}(NumVect\left(K_{q}\right) \end{array}\right\} \end{array}$$

The above leakage shows that the trapdoor is probabilistically encrypted. The encryption function of the trapdoor generation takes two arguments as input: one is a numerical representation of the plaintext query and the second is the public key.

(3) Leakage $L_{3}$: This leakage discusses the searching process evaluated over the cloud server. The cloud server performs the searching of phonemes and returns the relevant audio files. The cloud server can know about the returned file after searching but cannot know what we searched for. Leakage $L_{3}$ is defined as:

$$\begin{array}{c} L_{3}=\left\{\begin{array}{c} Enc_{R} \end{array}\right\} \end{array}$$

In the above equation, the *Enc*${}_{R}$ possesses the final search result in the encrypted form. The result is also probabilistically encrypted, so it does not leak information after searching.

NOTE: The definitions and leakages discussed above are aligned, verifying that the proposed scheme does not reveal any valuable information. In this way, we achieved privacy preservation.

### 6.2. Parameters Setting and Analysis

The performance results are based on parameters that Gentry defined in [37]. The author described the homomorphic evaluation of the advanced encryption standard (AES) using non-bootstrapping and bootstrapping implementation. The following are the security parameters on which security is based:

Q is the largest modulus, σ is the noise variance, while the security depends on the ratio of Q/σ.

$$N\geq \frac{\left((\log Q/\sigma )k+110\right)}{7.2}$$

where *N* is the dimension and this value depends upon Q/σ, which determines the security level.

For bootstrapping: the parameter sets are: *m* = 28,679, Φ(*m*) = 23,040. Using these parameters, Gentry achieved 123-bit security in 23 computations.

For non-bootstrapping: the parameter sets are: *m* = 53,261, Φ(*m*) = 46,080. Using these parameters, Gentry designed it to work on 40 computations. Setting such parameters was to achieve two AES rounds during the re-encryption phase. The computation and evaluation were performed by using a non-bootstrapping method. We performed operations, e.g., encryption and searching, using different values of *m* to analyze the different security levels. Table 3 shows the computation and evaluation results. The different plaintext file sizes are used. The table also shows that by increasing the security levels, the ciphertext file sizes are increased, thus increasing the search time as well.

## 7. Performance Analysis

In this section, the performance analysis is discussed, starting with the dataset generation, computational complexity, system specifications, and finally, the implementation results.

### 7.1. Dataset Generation

This research work utilizes the medical voice recordings dataset provided by medical speech, transcription, and intent [38]. The total data size was 11 GB. We generated five different sizes of phoneme files. Each phoneme file was composed of a different number of audio files. The audio files we took for the purpose were 50, 150, 200, 300, and 500. First, the audio files were converted to text, then converted to phonemes. The range of phonemes for a maximum of 500 files was around 5422. The details are discussed below with the performance results.

#### 7.1.1. Library for Audio to Transcribe

**Figure 3:**
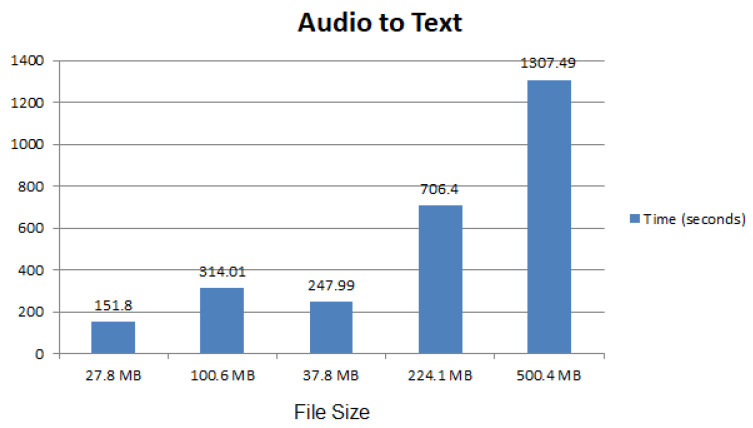
Audio to text conversion performance.

A subroutine was written in Python language based on Google speech recognition. This function adds multiple audio files and generates one text file, as shown in Figure 3. *x*-axis shows the file size in kilobytes while the y-axis shows the time in seconds. It was observed that with the increase in the file size, the time to convert audio to text also increased, as expected.

#### 7.1.2. Library for Text to Phonemize

**Figure 4:**
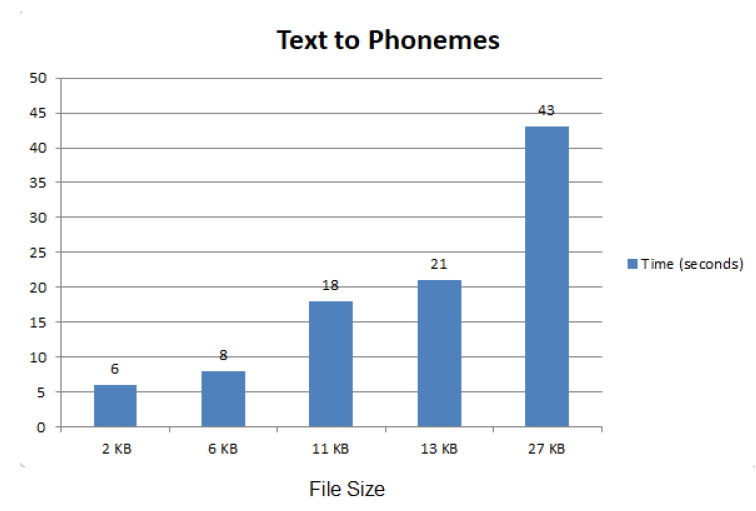
Text to phoneme conversion performance.

A Python subroutine was designed based on the Phonemize library [39]. It takes a single text file as input and generates one phoneme file, as shown in Figure 4. The x-axis shows the file size in kilobytes while the y-axis shows the time to convert the text to phonemes in seconds. With the increase in the file size, the time required for the conversion also increases.

### 7.2. Computational Complexity

In this section, the computational complexities of different cryptosystems, including HElib, which we used, are presented in Table 4. The time complexity of this scheme based on HElib is shown in Table 5 in terms of asymptotic notations for every algorithm. The setup phase was composed of initialization and key generation, and the time complexity was O(1). The encryption algorithm encrypts phonemes (*Ph*) in the number of audio files (N), the time complexity is O(NPh∗HE), where HE represents the complexity of HElib. The trapdoor generation algorithm simply encrypts the plaintext query input, so the time complexity is O(HE). The time complexity of the searching algorithm is $O(NT_{E})$ because it searches the query in all files (*N*). Similarly, the time complexity of decrypting search results and files is O(N∗HE).

### 7.3. Client-Side System Specification

The client’s side simulations were carried out on OS Ubuntu 18.04.5 LTS (64 bits) with 16 GB RAM, Intel Core i7-7700 CPU @ 3.6 GHz × 8, and 1 TB SSD storage, as shown in Table 6.

### 7.4. Cloud Side System Specification

The Contabo public cloud service was used to carry out our search phase on the server-side. The operating system was Ubuntu 20.04. The detailed client and server-side specifications are shown in Table 6.

### 7.5. HE Library by IBM

The operations for phoneme searching were carried out using HElib version 2.1.0 [35] in the Ubuntu operating system. HElib is an open-source programming library that carries out homomorphic encryption (HE). Brakerski–Gentry–Vaikuntanathan (BGV) was implemented in this scheme with bootstrapping; the Cheon–Kim–Kim–Song (CKKS) approximate number plan was used alongside numerous enhancements to make the homomorphic performance quicker. The ciphertext packing technique by Smart–Vercauteren and the optimization technique by Gentry–Halevi–Smart were also used. Since mid-2018, HElib has been under broad refactoring for reliability, robustness, serviceability, and performance. Above all, it supports developers and engineers working on homomorphic encryption.

### 7.6. Implementation

**Figure 5:**
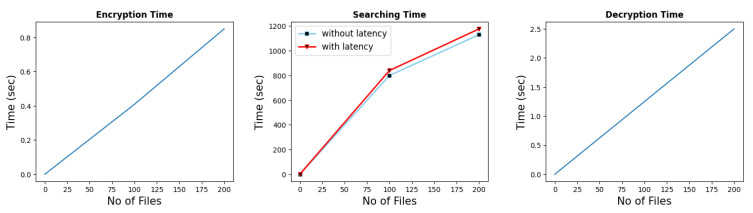
Results of multiple files, when *m* = 130 and zero security level.

**Figure 6:**
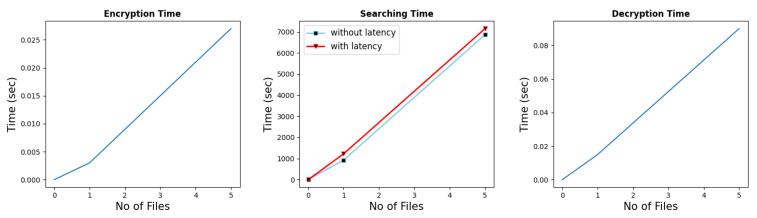
Results of multiple files, when *m* = 21,845 and 20.2167 security level.

This section elaborates on the performance of encryption, trapdoor generation, searching, and decryption. For implementation, the client–server architecture was followed. The encryption, query generation, and decryption were performed on the client-side, while the searching operation was performed on the cloud side. The final implementation was tested over a single file while scaling it to multiple files. The value of ‘*m*’ for the security level we considered was derived from the author Halevi in [42]. The author used the value of *m* = 21,845 while achieving the security level of 76. In this research, we also set *m* = 21,845 with *p* = 131 and we achieved a 20.2 security level while lattice dim ϕ(*m*) was 16,384. First, Figure 5 shows all operations while *m* = 130 and *p* = 131 for the dataset comprising 200 audio files with a negligible security level. The testing was done in batches of 25 files and the maximum number of files was 200. The x-axis shows the number of files and the y-axis shows the time in seconds. The result shows near linearity in performance. Since the search time was calculated on the cloud side and a response was sent back to the client-side, it possessed the network latency. Therefore, we also showed the difference in performance in terms of network latency. The small value of ‘*m*’ leaves a tiny polynomial ring, which is prone to several brute force attacks. Even without the secret key, guessing the extra error (including the point where it is introduced) would allow an adversary to decrypt the ciphertext. Similarly, the Figure 6, shows all the operations with a security level of 20.2, while the dataset comprised five audio files. The dataset was reduced because as we increase the security level, the search time increases, which is evident from the search time graph. The search time is again depicted while taking the latency into account. It can be observed that with the increase in the security level, the processing time also increases. Therefore, depending on the underlying use case and criticality of the data, one may choose the security level to be used. The number of files is presented along the *x*-axis; the time in seconds is presented along the *y*-axis.

## 8. Discussion

This section maps our proposed scheme against the design goals that were presented earlier in the introduction. In this paper, we proposed a scheme that allows a phonetic search over the encrypted audio data stored in the cloud. The encryption used was HElib, where only an authorized person is permitted to search over the encrypted data. Furthermore, we generated probabilistic trapdoors using FHE. It resists distinguishability attacks and search pattern hiding and achieves a privacy-preserving search. We introduced lightweight data structures to allow us to deploy the scheme in a real-world cloud environment, which has been demonstrated by deploying the scheme over a public cloud—Contabo. Table 7 summarizes our discussion and shows the description of how we achieved those goals.

## 9. Conclusions and Future Work

This paper presents a novel method of preserving the privacy of healthcare audio data stored in the cloud using fully homomorphic encryption. This research focuses on phonemes/audio searching over the cloud without decrypting the audio files. For this purpose, the proposed scheme utilizes a homomorphic encryption library (HElib). The library is probabilistic encryption with a BGV scheme. We achieved data privacy in the cloud while querying the phonemes and probabilistically searching the desired file. Thus, this paper addresses the security concerns regarding search and access patterns. The datasets utilized in this research work are real-world audio conversations between doctors and patients. Furthermore, we assessed different security levels and analyzed the scheme’s performance. Finally, the implementation of the client–server architecture was performed using multiple audio files while integrating the scheme with the public cloud platform "Contabo". The analysis shows that the computation time increases with increased security levels. This research work can be extended through multi-threading to increase the performance speed, using more audio files with high-security levels, and implemented using other open-source libraries, e.g., HEAAN, SEAL, and PALISADE. The performance can also be increased by using a cloud server with high specifications, automatically scaling up the resources as per the requirements.

## Figures and Tables

**Table 1:** Comparative analysis of different proposed schemes.

Paper	Technique Used	Confidentiality	HE	Searching Capability	Privacy Preservation
[11]	Newton-Raphson	✓	✓		✓
[12]	Neural Network	✓	✓	✓	✓
[13]	Index-based	✓		✓	✓
[31]	Blockchain	✓			
[32]	Attribute Encryption XACML	✓			✓
[16]	Hyper-ledger	✓			✓
[18]	Proxy Signature Group Signature	✓			✓
[15]	Blockchain	✓	✓		✓
[24]	Index-based	✓	✓	✓	
[25]	Index-based	✓	✓	✓	
[26]	DGHV HE	✓	✓	✓	
[27]	Index-based	✓			✓
[28]	Tree-based Index, Depth-First Search	✓		✓	✓
[33]	Public Encryption with Keyword Search (PEKS)	✓		✓	✓
[29]	Dual Word Embeddings, kNN Scheme	✓		✓	✓
Proposed	Fully HE	✓	✓	✓	✓

**Table 2:** Notations and descriptions.

Notation	Description
$S_{k}$	Secret key
$P_{k}$	Public key
λ	Security parameter for FHE
hwt	Hamming weight
p	Plaintext space modulus
m	Cyclotomic polynomial-defines phi(m), this will give a number of slots
r	Hensel lifting (default = 1)
bits	Number of bits of the modulus chain
c	Number of columns of Key-Switching matrix (default = 2 or 3)
$E_{Audio}$	Encrypted audio files
nthreads	Size of NTL thread pool (default =1)
$E_{Ph}$	Encrypted phonemes
$k_{q}$	Query
$Enc_{R}$	Result after searching phase
X	Plain text
*F*	Set of phoneme files
$N_{v}$	Numerical vector
N	Number of input files
Ph	Phonemes
C	Ciphertext
*Q*	Phonemes (plaintext) query
$T_{E}$	Encrypted trapdoor

**Table 3:** *m* parameter for different security levels.

Serial Number	1	2	3	4
‘m’ value	53,261	28,679	14,339	12,169
Security Level	93.77	36.8	12.63	9.22
Plaintext File Size (Bytes)	54	179	179	179
Number of Phonemes	10	30	30	30
Enc Time (sec)	1.19	1.63	0.8	0.77
Ciphertext File Size (MBs)	297.2	445.6	255.8	229.3
Search Query Time (sec)	2736	1880	110.7	174.7

**Table 4:** Computational complexity.

Basic Paillier (PPHE)	$O(|n^{3}|)$
CRT-PHES [40]	$O(|n^{2}||\alpha |)$
NC-PHES [41]	O(log(n))
HElib [35]	$O(nLlog^{2}p)$
DGHV	$O(n^{12})$

**Table 5:** Time Complexity of HElib.

Setup	O(1)
Encryption	O(NPh∗HE)
Trapdoor Generation	O(HE)
Searching	$O(NT_{E})$
Decryption	O(N∗HE)

**Table 6:** Specifications.

Specifications	Contabo Cloud	Client
OS	Ubuntu 20.04	Ubuntu 20.04 (64 bits)
CPU Cores	10 vCPU Cores	Intel i7-7700 CPU @ 3.6 GHz × 8
RAM	60 GB	16 GB
Storage	1.6 TB	1 TB SSD
Network speed	1 Gbit/s	100 MB/s

**Table 7:** Security goals description.

Security Goals	Implementation Description
Data confidentiality	Is achieved by homomorphically encrypting the telemedicine data.
Searching capability	By presenting a fully homomorphic encryption searching scheme.
Authorized person searching	Only an authorized person in possession of the correct cryptographic keys can generate a search query and decrypt the files.
Privacy-preserving	Probabilistic encryption is introduced, preserving the privacy of the data.
Search pattern hiding	The trapdoors are probabilistic, achieving search pattern hiding.
Cloud deployable	The proposed scheme is implemented and tested over the Contabo CSP

## Data Availability

Not applicable.
